# Clinical Instruction at Chicago

**Published:** 1847-06

**Authors:** 


### PART V.—EDITORIAL.

### ARTICLE I.

### CLINICAL INSTRUCTION AT CHICAGO.

In order to enable our readers to judge of the facilities for obtaining a practical knowledge of medicine and surgery in the medical college at Chicago, we give below a table of the cases prescribed for at the College Dispensary, and the surgical cases treated at the Hospital from November 15th, 1846, up to May 15th, 1847, together with the operations performed in presence of the class during that time. In addition to these, a considerable number have been attended at their own houses by the more advanced students. The hospital is at present in successful operation, and the dispensary is attached to it. Great want of a suitable building is, however, felt, that at present occupied being but temporarily used for that purpose. This want will, however, soon be remedied, as the public authorities have in contemplation the erection of a suitable edifice for the accommodation of all classes of patients.

*Table of Cases treated at the College Dispensary at Chicago, from its commencement, November 15th, to May 15th, 1847.*

| | | | |
|---|---|---|---|
| Dyspepsia | 5 | Pneumonia | 1 |
| Hypertrophy of the Heart | 2 | Catarrh | 6 |
| Valvular Disease of the Heart | 3 | Dislocation of Shoulder | 1 |
| | | Deformed Cicatrix | 1 |
| Dilatation of the Heart | 1 | Quinsy | 1 |
| Enlarged Tonsils | 1 | Ptosis | 1 |
| Ophthalmia | 6 | Phthisis | 1 |
| Dislocation of the Elbow | 2 | Wound of Eyes | 1 |
| Indolent Ulcers | 12 | Asthma | 4 |
| Abscess | 6 | Elongated Uvula | 1 |
| Anasarca | 8 | Phymosis | 1 |
| Chronic Pleurisy | 1 | Rheumatism | 6 |
| Nasal Polypi | 2 | Constriction of Larynx | 1 |
| Caries | 4 | Porrigo | 1 |

| | | | |
|---|---|---|---|
| Neuralgia | 6 | Vertigo | 1 |
| Strabismus | 2 | Infantile Remittent | 5 |
| Diseased Stump | 2 | Dentition | 4 |
| Dysmenorrhœa | 2 | Goitre | 1 |
| Gastritis | 1 | Constipation | 3 |
| Necrosis | 3 | Diarrhœa | 2 |
| Peritonitis | 2 | Schirrhus Tumor | 1 |
| Hernia | 1 | Encysted Tumor | 3 |
| Bronchitis | 9 | Prurigo | 2 |
| Hysteria | 1 | Cephalalgia | 2 |
| Scabies | 5 | Ptyalism | 1 |
| Inscised Wound | 4 | Ulcerated Sore-throat | 1 |
| Encysted Tumour | 3 | Croup | 1 |
| Erysipelas | 1 | Nephritis | 1 |
| Morbus Coxarius | 1 | Ecthyma | 1 |
| Fracture of Metacarpal Bones | 3 | Hare-lip | 1 |
| | | Club-foot | 4 |
| Fracture of Femur | 1 | Abdominal Tumor | 1 |
| Fracture of Neck of Femur | 1 | Schirrhus Pylorus | 2 |
| Fracture of Radius | 2 | Vicarious Menstruation | 1 |
| Fracture of Tibia and Fibula | 2 | Fistula in Ano | 2 |
| | | Dislocation of Elbow | 2 |
| Fracture of Clavicle | 1 | Polypus of External Ear | 1 |
| Influenza | 1 | Cutaneous Cancer | 1 |
| Intermittent Fever, with its attendants and sequelæ | | | 202 |
| Total | | | 379 |

Most of these returned a number of times so that the course of the disease and the effects of treatment could be watched.

The following operations, dressings, &c., have been performed.

| | |
|---|---|
| Excision of Tonsils, | 1 |
| For Fistula in Ano by division, | 2 |
| For Fistula in Ano by ligature, | 1 |
| Extirpation of Encysted Tumors, | 3 |
| Extirpation of Scirrhus Tumors, | 1 |
| Operation for Hare-lip, | 1 |
| Amputation of Finger, | 1 |

| | |
|---|---|
| Amputation of the Metatarsal Bones, - - | 1 |
| Operations for Club-foot, - - - - - | 4 |
| Operation for Ptosis, - - - - - - | 1 |
| Operations for Trichiasis, - - - - - | 2 |
| Operation for Cutaneous Cancer, - - | 1 |
| Extraction of Nasal Polypi, - - - | 3 |
| Operation for Strabismus, - - - - | 1 |
| Removal of Vicious Cicatrix, - - | 1 |
| Removal of Polypus of the Ear, - - - | 1 |
| Operation for Phymosis, - - - - | 1 |
| Extirpation of Abdominal Tumor, - - | 1 |
| Reduction and Dressing of Dislocation of Elbow of five months' standing, - - | 1 |
| Reduction and dressing of Fracture of Metacarpal Bones, - - - - - | 3 |
| Reduction and Dressing Fracture of Radius, | 2 |
| Reduction and Dressing Fracture of Femur, | 1 |
| Reduction and Dressing Fracture of Tibia and Fibula - - - - - | 2 |
| Reduction and Dressing Fracture of Clavicle, | 1 |
| Total - - - - - | 37 |

*Physicians and Surgeons to the Chicago Hospital.*

Attending Physician—Dr. H. S. Huber.

Consulting Physicians—Dr. E. S. Kimberly,
Dr. J. Brinckerhoff,
Dr. G. D. Boone,
Dr. J. V. Z. Blaney.

Attending Surgeon—Dr. D. Brainard.

Consulting Surgeons—Dr. W. B. Herrick,
Dr. P. Maxwell.

